# Alcohol Consumption and the Risk of Incident Atrial Fibrillation: A Meta-Analysis

**DOI:** 10.3390/diagnostics12020479

**Published:** 2022-02-13

**Authors:** Georgios Giannopoulos, Ioannis Anagnostopoulos, Maria Kousta, Stavros Vergopoulos, Spyridon Deftereos, Vassilios Vassilikos

**Affiliations:** 1 3rd Department of Cardiology, Medical School, Aristotle University of Thessaloniki, Hippocration General Hospital, 546 42 Thessaloniki, Greece; stavverg@gmail.com (S.V.); vvassil@auth.gr (V.V.); 2 Department of Cardiology, Athens General Hospital “G. Gennimatas”, 115 27 Athens, Greece; iannis.anagnostopoulos@gmail.com (I.A.); maria.s.kousta@hotmail.com (M.K.); 3 2nd Department of Cardiology, Medical School, National and Kapodistrian University of Athens, 157 72 Athens, Greece; spdeftereos@gmail.com

**Keywords:** atrial fibrillation, incidence, alcohol, risk factor, drinking, lifestyle

## Abstract

Alcohol consumption is a known, modifiable risk factor for incident atrial fibrillation (AF). However, it remains unclear whether the protective effect of moderate alcohol consumption-that has been reported for various cardiovascular diseases also applies to the risk for new-onset AF. The purpose of this meta-analysis was to evaluate the role of different drinking patterns (low: <14 grams/week; moderate: <168 grams/week; and heavy: >168 grams/week) on the risk for incident AF. Major electronic databases were searched for observational cohorts examining the role of different drinking behaviors on the risk for incident AF. We analyzed 16 studies (13,044,007 patients). Incident AF rate was 2.3%. Moderate alcohol consumption significantly reduced the risk for new-onset AF when compared to both abstainers (logOR: −0.20; 95%CI: −0.28–−0.12; I2: 96.71%) and heavy drinkers (logOR: −0.28; 95%CI: −0.37–−0.18; I2: 95.18%). Heavy-drinking pattern compared to low also increased the risk for incident AF (logOR: 0.14; 95%CI: 0.01–0.2; I2: 98.13%). Substantial heterogeneity was noted, with more homogeneous results documented in cohorts with follow-up shorter than five years. Our findings suggest a J-shaped relationship between alcohol consumption and incident AF. Up to 14 drinks per week seem to decrease the risk for developing AF. Because of the substantial heterogeneity observed, no robust conclusion can be drawn. In any case, our results suggest that the association between alcohol consumption and incident AF is far from being a straightforward dose-response effect.

## 1. Introduction

Atrial fibrillation (AF) is the most common arrhythmia in adults [1], with an estimated prevalence between 2% and 4% [2]. The complexity of its pathogenesis requires a holistic and multidisciplinary approach to the management of AF patients, and the potential impact of multiple comorbidities on AF risk underscores the importance of controlling modifiable risk factors.

Modifiable risk factors are potent contributors to AF development and progression [3]. The Atrial Fibrillation Better Care (ABC) pathway provides an integrated model of care of AF patients as compared to usual care [4,5]. The “C” part of the ABC pathway involves, among other interventions, investigation and management of unhealthy lifestyle factors, such as smoking, alcohol consumption, and physical inactivity [6]. Specifically, both acute and chronic alcohol consumption are modifiable risk factors for incident AF [7,8]. Moreover, a recent RCT reported that alcohol abstinence reduced AF recurrence in regular drinkers with AF [9]. Nevertheless, whether there is a kind of protective alcohol consumption, as it has been reported for a variety of cardiovascular diseases [10], as well as the amount of alcohol that may be clinically relevant [11] remain unclear.

In this systematic review and meta-analysis, we aimed to explore the impact of different drinking patterns on the risk for developing AF.

## 2. Materials and Methods

This systematic review and meta-analysis was performed according to the PRISMA guidelines [12]. The predefined protocol was registered in PROSPERO database (ID: 303961).

Medline (via PubMed) and Scopus were searched using a strategy based on the following combination of keywords (((“alcohol consumption”) OR (“alcohol drinking”) OR (“binge drinking”) OR (“alcohol intake”)) AND (“atrial fibrillation”)) from inception up to December 2021. Additional hand-search was also performed using the references of the articles that were identified as relevant (snowball strategy).

After deduplication, two independent reviewers (M.K. and I.A.) screened all articles at title and abstract level. Potentially eligible studies were further reviewed based on the full text. In the final analysis, we included observational cohorts examining the association between alcohol consumption and incident AF, if they had a follow-up period of at least 1year, and they reported raw incidence numbers (cases/controls) for at least 3 categories of drinking behavior (i.e., abstainers/low consumption, moderate consumption, and heavy consumption). Articles not available in English were excluded. Any disagreements were resolved by consultation with an expert (G.G.).

Data regarding studies and patient characteristics as well as the outcome of interest were extracted in a predesigned Microsoft Office Excel 2007 by two independent reviewers (M.K. and I.A.). Subsequently, they were crosschecked for any disagreements, which were resolved by a senior (G.G.). Alcohol consumption categories are summarized as grams of alcohol per week (gr/w). When categories were expressed as grams per day, they were transformed to gr/w by multiplying with 7. If they were expressed as standard drinks or units, we assumed that each standard drink contains 12 g of alcohol, and every unit equals 8 g of pure ethanol, according to the definitions mostly used in the included studies. When more than 3 categories were available, we tried to combine them into meaningful groups. We defined abstainers/low consumption as 0–14 gr/w, moderate consumption <168 gr/w (or <84 gr/w for women), and heavy consumption as >168 gr/w (or >84 gr/w for women).

Risk of bias within studies was assessed in duplicate with the National Institutes of Health Quality Assessment Tool for Observational Cohort and Cross-Sectional Studies [13]. Disagreements were resolved by consensus. Studies with an overall score ≥80% were deemed as high quality. Publication bias was evaluated both visually, using contour-enhanced funnel plots (with the level of statistical significance set at 1%, 5%, and 10%) and with Egger’s test [14].

Statistical analysis: Continuous variables were summarized as mean (standard deviation). For pooling the outcomes of interest, we first calculated odds ratios (OR) along with their corresponding 95% confidence intervals (CI); subsequently, we transformed logarithmically the calculated OR and 95%CI to perform the final analysis. To allow for expected effect size dispersion between studies, a random effects (DerSimonian–Laird) model was adopted. Heterogeneity was assessed using I^2^, with values more than 50% representing substantial heterogeneity [15]. To investigate the heterogeneity observed, we performed subgroup analysis based on the duration of follow up and the region that each study was conducted and sensitivity analysis, including only high-quality studies. Furthermore, meta-regression analysis was used to investigate the confounding effect of mean age, mean body mass index, number of males, smokers, and hypertensive and diabetic patients. Finally, we evaluated the linearity between weekly alcohol consumption and the risk for AF. A restricted cubic spline model, with three knots at 25%, 50%, and 75% of the distribution, was crated (using the original subcategories of alcohol intake per week). Linearity was assessed by comparing the slopes of the regression line using the Wald test, with *p*-values < 0.05 indicating nonlinearrelationship [16]. All analyses were performed using STATA/MP version 16.0, Texas, USA, and R (R Foundation, version 3.6.3) softwares.

## 3. Results

**Figure 1:**
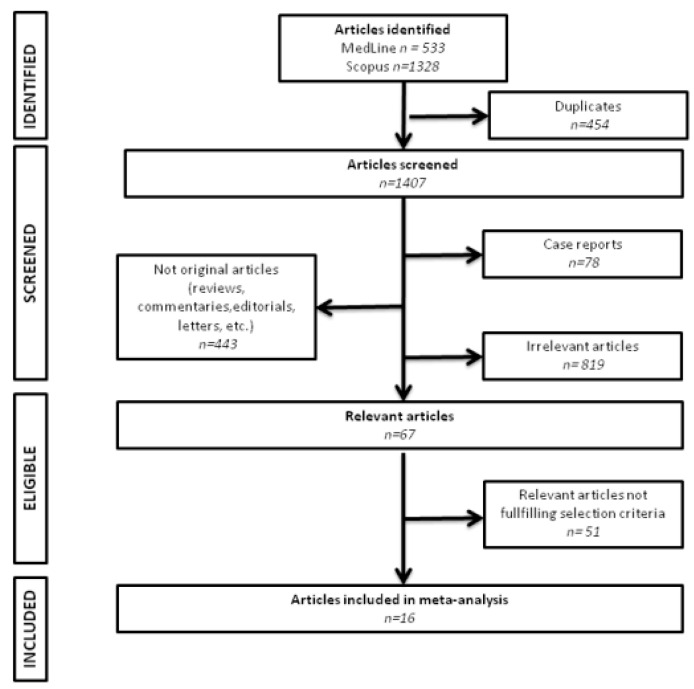
Flow of study selection.

We identified 19 eligible studies. Three of them were excluded due to patient overlapping; thus, we analyzed 16 cohorts [8,17,18,19,20,21,22,23,24,25,26,27,28,29,30,31]. The flow of study selection is summarized in Figure 1. In total, 13,044,007 patients were analyzed, and 305,433 (2.3%) cases of incident AF were documented. Most of the studies were conducted in Europe, and the overall follow-up ranged from 2.7 to up to 50 years. AF was mostly ascertained using medical records and the International Classification of Diseases (ICD) system. Five studies used periodic electrocardiograms to identify patients with newly diagnosed AF. Data regarding the incidence of AF in the low-consumption category were available in all studies, while 15 and 14 of them provided meaningful information for the moderate and heavy-consumption categories, respectively. Study characteristics are summarized in Table 1. Transformations used to estimate weekly alcohol consumption in each study are depicted in Supplementary Table S1.

The risk of bias assessment is summarized in Supplementary Table S2. Nine studies were deemed as moderate quality (score between 70 and 80%), mainly due to the absence of both power analysis description and exposure reassessment. Only four cohorts presented low risk of bias. No significant publication bias was revealed except the analysis regarding moderate versus low consumption. (Supplementary Figures S1–S3). The corresponding Egger’s test *p*-values were 0.08 for heavy versus low consumption, <0.001 for moderate versus low consumption, and 0.9 for heavy versus moderate consumption.

**Figure 2:**
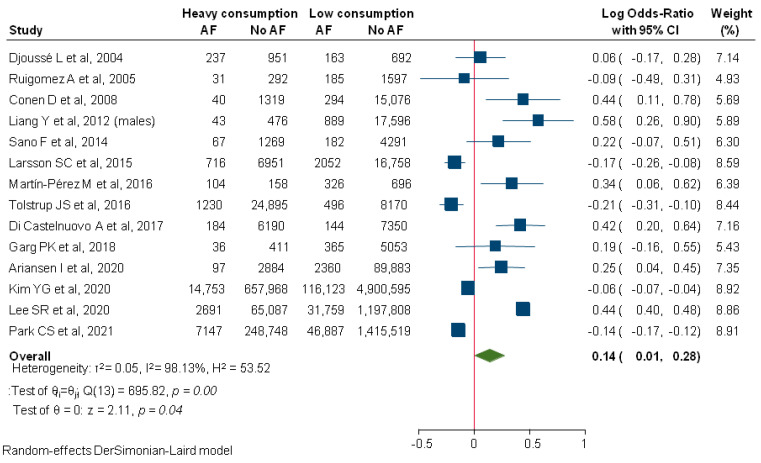
Comparison between heavy and low alcohol consumption regarding incident AF. AF, Atrial Fibrillation.

Compared to low consumption, heavy alcohol consumption significantly increased the risk for new-onset AF (logOR: 0.14; 95%CI: 0.01–0.2; I2: 98.13%; Figure 2). In subgroup analysis, significantly lower heterogeneity was documented for studies with an overall follow up of less than 5 years (logOR: 0.37; 95%CI: 0.17–0.56; I2: 62.23%), while the effect size was non-significant for studies that followed patients for a longer time period (logOR: 0.07; 95%CI: −0.06–0.19; I2: 87.27%).

**Figure 3:**
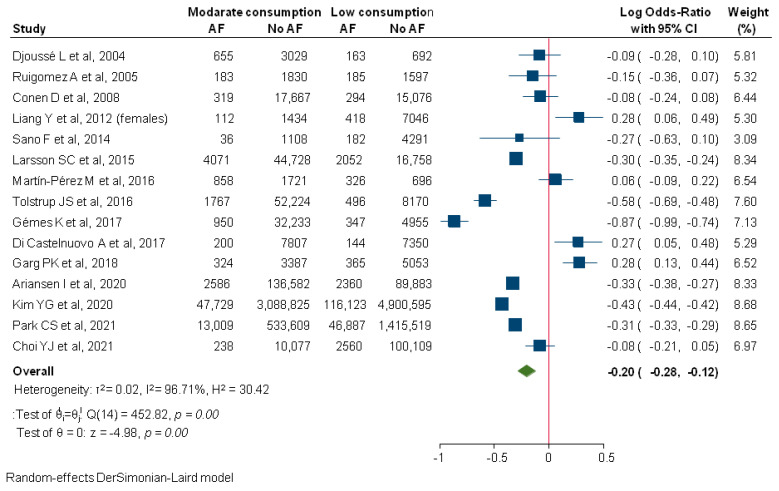
Comparison between moderate and low alcohol consumption regarding incident AF. AF, Atrial Fibrillation.

Patients following a moderate consumption pattern were at significantly lower risk for new-onset AF compared to abstainers (logOR: −0.20; 95%CI: −0.28–−0.12; I2: 96.71%; Figure 3). An increasing number of hypertensive patients (*p* = 0.04) significantly augmented the effect size estimation in meta-regression analysis, explaining almost 10% of the initial heterogeneity.

**Figure 4:**
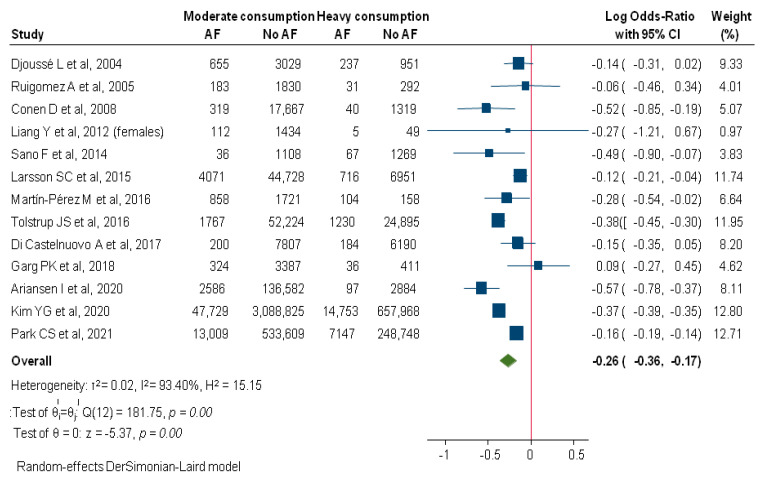
Comparison between moderate and heavy alcohol consumption regarding incident AF. AF, Atrial Fibrillation.

When compared to the heavy-drinking pattern, moderate alcohol consumption was also associated with decreased incidence of AF (logOR: −0.26; 95%CI: −0.36–−0.17; I2: 93.4%; Figure 4). Higher mean age of the included subjects significantly decreased this protective effect (*p* = 0.03), explaining 43% of the initial heterogeneity. Moreover, analysis of the subgroup of studies with shorter follow up (<5 years) revealed a highly homogeneous estimation (logOR: −0.22; 95%CI: −0.43–−0.003; I2: 0%), while decreased but substantial heterogeneity was found for studies with longer follow up (logOR: −0.25; 95%CI: −0.36–−0.15; I2: 84.61%).

Sensitivity analysis based on four high-quality cohorts did not change the above-mentioned results (Supplementary Figures S4–S6).

**Figure 5:**
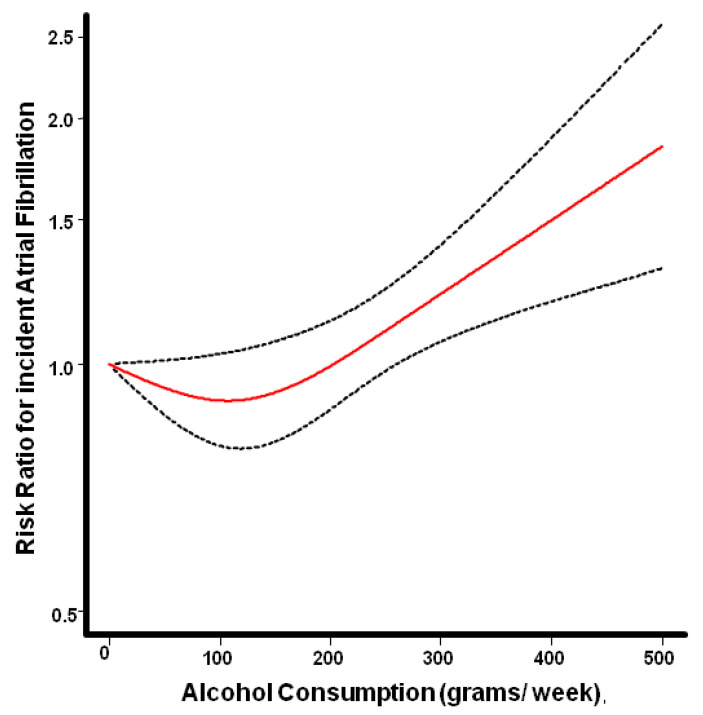
Pooled dose response relationship between weekly alcohol consumption and risk for incident atrial fibrillation seems to follow a J shaped pattern. Abstinence served as the reference category. The red line depicts the estimated risk ratio, while the dotted lines represent the corresponding 95% confidence intervals.

Regression analysis, using the restricted cubic spline model, demonstrated a J-shaped association between alcohol consumption and the risk for AF, without evidence of linearity (*p* = 0.0017), Figure 5.

## 4. Discussion

In this systematic review and meta-analysis, we assessed the potential association between alcohol consumption and incident AF. After categorizing weekly drinking behavior into low, moderate, and heavy drinking, we demonstrated that moderate alcohol consumption pattern may yield a kind of protective effect against new-onset AF. Subjects consuming up to two drinks per day were in significantly lower risk compared to heavy drinkers, especially hypertensive ones. Moreover, this attenuated risk of mild drinking pattern was maintained in the comparison with the abstainers although it seems to be more evident in younger subjects. The heavy-drinking pattern was found to be the most harmful, as it also significantly increased the risk for incident AF when compared to the low pattern. These results should be interpreted with great caution because of the substantial heterogeneity that was noted in all analyses. Comparisons involving the heavy-drinking pattern, in particular, seem to be more homogeneous in the subgroup of studies with shorter mean follow up, which might reflect changing drinking behavior over the time.

These results are in line with previous analyses reporting that high levels of alcohol intake increase the risk for new-onset AF [32,33,34]. Gallagher et al. [32] examined the impact of moderate drinking behavior. They reported that consuming up to 6–7 standard drinks per week is not associated with higher incidence of AF. The current analysis suggests that consuming up to 14 drinks per week is probably associated with a kind of “protective” effect against AF even when compared to an abstinence pattern (up to 1–2 drinks per week). This contradicts the conclusions of Larsson et al. [21], which suggested that even moderate alcohol consumption is a risk factor for atrial fibrillation. Their meta-analysis, however, differs significantly in terms of methodology and study selection.

Beneficial cardiovascular effects of moderate alcohol consumption have been previously described. Ding et al. recently reported that a reduction in both cardiovascular events and cardiovascular mortality was observed in patients with a weekly intake of 42–56 g of alcohol [10], while Yoon et al., despite almost similar results, questioned these protective effects in younger people and in patients with multiple comorbidities [35]. Moreover, various researchers have reported that light to moderate consumption either reduces or does not affect the incidence of various classic risk factors for AF development. Attenuated risk for type 2 diabetes [36], heart failure [37] and coronary heart disease [10,38] has also been described. Similar results have been documented for chronic kidney disease [39], chronic obstructive pulmonary disease [40], and mental health [41]. Finally, the role of alcohol consumption regarding incident hypertension remains controversial [42,43]. Conceptually, the notion that a factor, namely low-to-moderate alcohol consumption, which appears to have a largely beneficial effect on cardiovascular disease risk and even on overall mortality (25% lower mortality [6]), is straightforwardly detrimental in terms of incident AF borders on the paradoxical.

On the other hand, alcohol consumption seems to yield an unfavorable effect on atrial structure and function. Voskoboinik et al., after performing high-density mapping in patients undergoing AF ablation, reported lower bipolar voltages and more frequent complex potentials in drinkers compared to non-drinkers [44]. In addition, impaired left atrium mechanics [45] and increased levels of atrial natriuretic peptides [46] and left atrial size [47,48] have been documented in patients consuming alcohol regularly, irrespective of the amount.

This study has several limitations. A major one is the substantial heterogeneity that was found across all comparisons and limits the applicability of our findings. Furthermore, information about alcohol consumption was provided by the subjects, which may be a source of recall bias. It is also possible that a substantial number of patients might have changed their drinking behavior throughout the follow up. Moreover, baseline comorbidities, which could account for at least a part of the observed heterogeneity, were inadequately reported by most studies. Finally, the beverage-specific effects on incident AF may have influenced our analysis.

## 5. Conclusions

In this systematic review and meta-analysis, we demonstrate that the association between alcohol consumption and the risk for AF may follow a J-shaped curve; meta-regression analysis did not show that this pattern could be attributed to potential confounders. Based on current literature, this risk reduction might be attributed to the protective effect of moderate drinking regarding various known risk factors for AF development. These results should be interpreted with caution because of the substantial heterogeneity that was revealed, and no clear clinical implications can be suggested. Further studies emphasizing on the role of moderate consumption are needed to clarify this relationship and evolve our understanding regarding the underlying pathophysiology. In any case, our results suggest that the association between alcohol consumption and incident AF is far from being a straightforward dose-response effect.

## Figures and Tables

**Table 1:** Characteristics of the included studies.

Study	Region	Follow-Up (Years)	Method of AF Diagnosis	N	Males (%)	Age	BMI	HT (%)	DM (%)	Sm (%)
Djoussé L et al., 2004	US	>50	ECG	5727	51	45.8 8.04	NA	NA	12	NA
Ruigómez A et al., 2005	UK	2.7	ICD	5525	47.2	NA	NA	19.1	4.2	29.7
Conen D et al., 2008	U.S.	12.4	ECG. medical records	34.715	0	53.1 7.1	24.9 4.5	25.3	2.4	48.5
Liang Y et al., 2012	Multi-center	4.6	ECG	30.433	70	66.4 7.2	NA	70	37.2	62.3
Sano F et al., 2014	Japan	6.4	ECG. medical records	8284	35.7	56	NA	30.2	NA	18.6
Larsson SC et al., 2015	Sweden	12	ICD. ECG	75.276	58.2	60.5	25.4	22.3	7	23.9
Martín-Pérez M et al., 2016	UK	2.7	medical records	4489	55	NA	NA	NA	NA	58.7
Tolstrup JS et al., 2016	Denmark	6.1	ICD	88.782	45.1	57.4 14.5	25.4 4.1	18.1	3.7	22.3
Gémes K et al., 2017	Norway	8	ECG	47.002	44.9	52.3 15.7	27.1 4.4	NA	NA	55.9
Di Castelnuovo A et al., 2017	Italy	8.2	medical records	22.065	48.6	55.3 11.9	28 4.6	55.7	9.2	23.6
Garg PK et al., 2018	U.S.	9.4	ECG. self-reports	9576	42.6	63.3 8.1	29 6	NA	NA	12.6
Ariansen I et al., 2020 (males)	Norway	9	hospital discharge diagnosis	234.392	48.4	43.5 10	25.6 3.8	NA	1.2	63.4
Kim YG et al., 2020	Korea	NA	ICD	9.776.956	54.7	47 14.1	23.7 3.2	25.4	8.6	40.4
Lee SR et al., 2020	Korea	5	ICD	1.719.401	46	66 0	24.3 3	53	20.5	30.6
Park CS et al., 2021	Korea	7.1	ICD	2.551.036	59.9	57.7 11.9	NA	56.8	100	44.1
Choi YJ et al., 2021	Korea	4	ICD	112.984	35	63.3 10.6	25.1 3.3	21.7	100	23.8

AF, atrial fibrillation; N, number of patients; BMI, body mass index; HT, hypertension; DM, diabetes mellitus; Sm, smokers; NA, not available; U.S., United States of America; UK, United Kingdom; ECG, electrocardiogram; ICD, International Classification of Diseases. Continuous variables are summarized as mean (SD).

## Data Availability

Not applicable.

## Supplementary Materials

The following supporting information can be downloaded at: https://www.mdpi.com/article/10.3390/diagnostics12020479/s1, Table S1: Transformation of the original alcohol consumption categories for the final analysis. Table S2: Quality assessment, Figure S1: Contour enhanced funnel plot for the comparison between heavy and low alcohol consumption. Figure S2: Contour enhanced funnel plot for the comparison between moderate and heavy alcohol consumption. Figure S3: Contour enhanced funnel plot for the comparison between moderate and low alcohol consumption. Figure S4: Comparison between heavy and low alcohol consumption regarding incident AF. Sensitivity analysis. Figure S5: Comparison between moderate and heavy alcohol consumption regarding incident AF. Sensitivity analysis. Figure S6: Comparison between moderate and low alcohol consumption regarding incident AF. Sensitivity analysis.
