# Supplementary material for: Alcohol Consumption and the Risk of Incident Atrial Fibrillation: A Meta-Analysis
Source: Diagnostics (Basel). 2022 Feb 13;12(2):479. doi: 10.3390/diagnostics12020479 (PMC8871230; doi:10.3390/diagnostics12020479)
Supplement: Supplementary file 1 [file diagnostics-12-00479-s001.zip › diagnostics-1581397-supplementary.pdf]

Supplementary Table S1 Transformation of the original alcohol consumption categories for the final analysis.

Study	Category	Original cohort	Included in analysis
Djoussé L etal. 2004	Low	0 gr/d	0 gr/w
	Moderate	0.1-24gr/d	0.1-168gr/w
	Heavy	>24.1gr/d	>168gr/w
Ruigómez A etal. 2005	Low	0U/w	0-8gr/w
	Moderate	1-21U/w	8-168gr/w
	Heavy	>21U/w	>168gr/w
Conen D etal. 2008	Low	0SD/d	0gr/w
	Moderate	<1 &<2SD/d	0.1-168gr/w
	Heavy	>2SD/d	>168gr/w
Liang Y et al. 2012	Low	<1SD/w	0gr/w
	Moderate	♀1-14SD/w<br>♂1-21SD/w	♀0.1-168gr/w<br>♂0-210*
	Heavy	♀>14SD/w<br>♂>21SD/w	♀>168gr/w<br>♂>210gr/w
Sano F etal. 2014	Low	0gr/d	0gr/w
	Moderate	<23gr/d	0.1-161gr/w
	Heavy	>23gr/d	>161gr/w
Larsson SC etal. 2015	Low	0SD/w &<1SD/w	0-12gr/w
	Moderate	1-14SD/d	12.1-168gr/w
	Heavy	>15SD/d	>180gr/w
Martín-Pérez M et al. 2016	Low	0U/w	0-8gr/w
	Moderate	1-20U/w	8-160gr/w
	Heavy	>21U/w	>168gr/w
Tolstrup JS etal. 2016	Low	<1SD/w	0-12gr/w
	Moderate	1-13 SD/w	12.1-156gr/w
	Heavy	>14SD/w	>168gr/w
Gémes K etal. 2017	Low	Abstainers	0gr/w
	Moderate	0-7SD/w	0.1-84gr/w
	Heavy	>7SD/w	>84gr/w*
Di Castelnuovo A et al. 2017	Low	0gr/d or <1gr/d	0-7gr/w
	Moderate	1-24gr/d	7-168gr/w
	Heavy	>24gr/d	>168gr/w
Garg PK etal. 2018	Low	None	0gr/w
	Moderate	♀1SD/d<br>♂1-2SD/d	♀0.1-84gr/w<br>♂0-168gr/w
	Heavy	♀>1SD/d<br>♂>2SD/d	♀>84gr/w<br>♂>168gr/w
Ariansen I etal. 2020	Low	<2gr/d	<14gr/w
	Moderate	2-24gr/d	14-168gr/w
	Heavy	>24gr/d	>168gr/w
Kim YG etal. 2020	Low	0gr/w	0gr/w
	Moderate	0-105gr/w	0-105gr/w
	Heavy	>210gr/w	>210gr/w
Lee SR etal. 2020	Low	0	0gr/w
	Moderate	<30gr/d	0.1-210gr/w
	Heavy	>30gr/d	>210gr/w
Sterling SA etal. 2020	Low	0	0gr/w
	Moderate	♀<3SD/d - <7SD/w<br>♂<4SD/d- <14SD/w	
	Heavy	♀>3SD/d - >7SD/w<br>♂>4SD/d- >14SD/w	♀>84gr/w<br>♂>168gr/w

Park CS etal. 2021	Low<br>Moderate<br>Heavy	0<br><15gr/d<br>>30gr/d	0gr/w<br>0.1-105gr/w<br>>210gr/w
Choi YJ etal. 2021	Low<br>Moderate<br>Heavy	0<br><20gr/d<br>20>gr/d	0gr/w<br>0.1-140gr/w<br>>140gr/w

gr: grams, d: day, w: week, U: units, SD: standard drinks

Supplementary Table S2 Quality assessment

Study	1	2	3	4	5	6	7	8	9	10	11	12	13	14	Overall
Djoussé L et al. 2004	2	2	2	1	0	1	2	2	2	2	2	1	2	2	23
Ruigómez A et al. 2005	2	2	2	2	0	0	1	2	1	0	2	0	2	2	18
Conen D et al. 2008	2	2	2	2	0	2	1	2	2	2	2	1	2	2	24
Liang Y et al. 2012	2	2	2	2	0	2	1	2	2	0	1	1	2	2	21
Sano F et al. 2014	2	2	0	2	0	2	2	2	2	0	1	1	2	2	20
Larsson SC et al. 2015	2	2	2	2	0	2	2	2	2	0	1	1	2	2	22
Martín-Pérez M et al. 2016	1	2	2	2	0	0	1	2	1	0	2	0	2	2	17
Tolstrup JS et al. 2016	2	2	1	0	0	2	1	2	2	1	2	1	2	2	20
Gémes K et al. 2017	2	2	2	2	0	2	2	2	2	0	1	2	2	2	22
Di Castelnuovo A et al. 2017	2	2	2	2	0	2	2	2	2	0	1	1	2	2	22
Garg PK et al. 2018	2	2	2	2	0	2	2	2	1	2	2	1	0	2	22
Ariansen I et al. 2020	2	2	2	2	2	2	2	2	2	0	2	1	2	2	25
Kim YG et al. 2020	2	2	2	2	0	2	2	2	2	0	2	1	2	2	23
Lee SR et al. 2020	2	2	2	2	0	2	1	2	2	0	1	1	2	2	21
Park CS et al. 2021	2	1	2	2	0	2	1	2	1	0	1	1	2	2	19
Choi YJ et al. 2021	2	2	1	2	0	2	1	2	2	0	1	1	2	2	20

Supplementary Figure S1 Contour enhanced funnel plot for the comparison between heavy and low alcohol consumption.

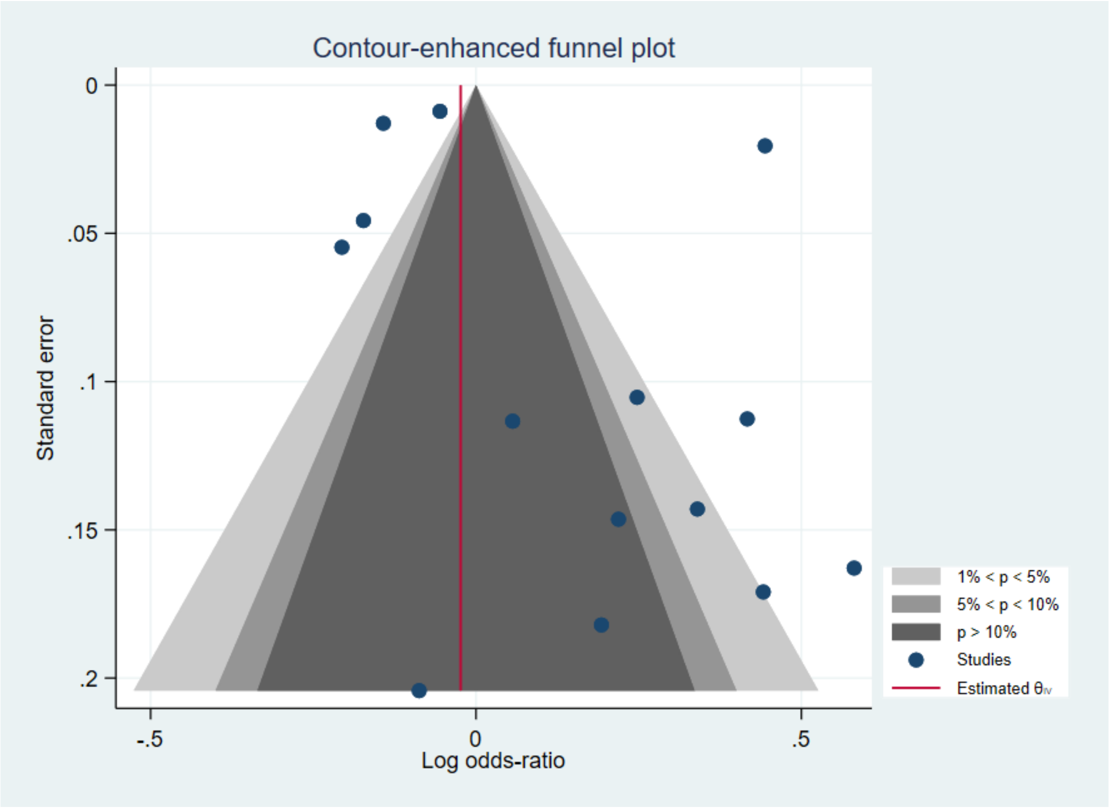

Supplementary Figure S2 Contour enhanced funnel plot for the comparison between moderate and heavy alcohol consumption.

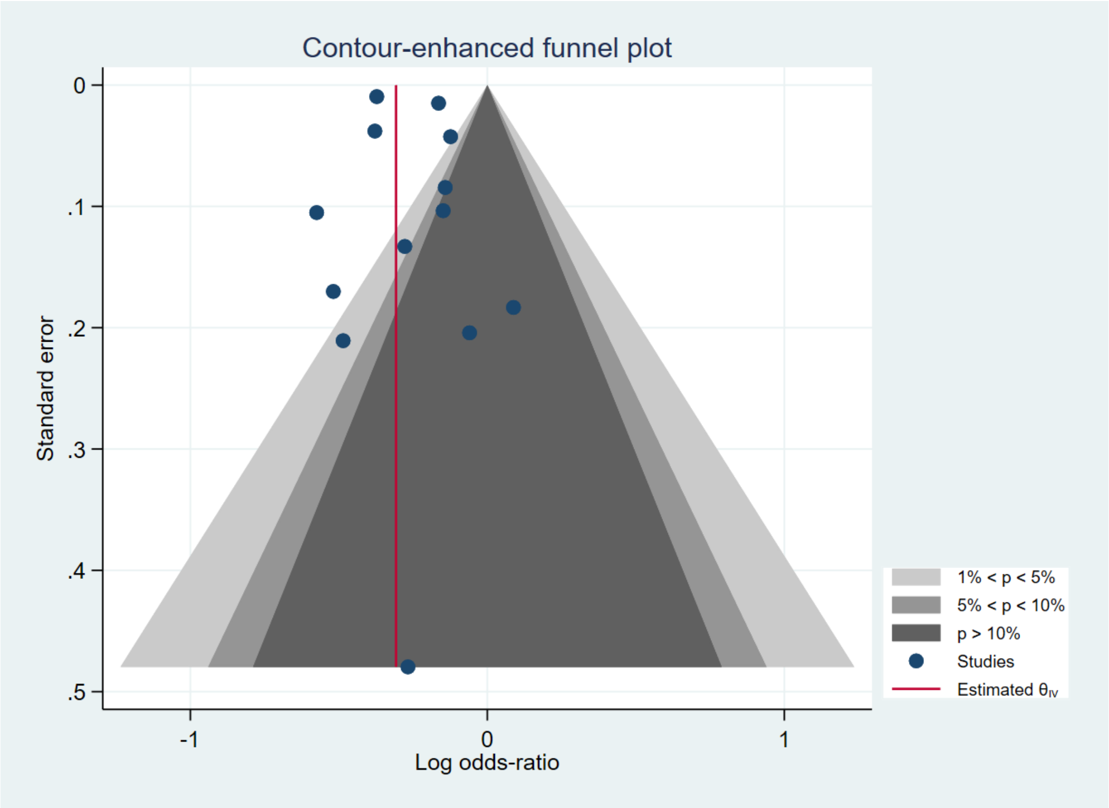

Supplementary Figure S3 Contour enhanced funnel plot for the comparison between moderate and low alcohol consumption.

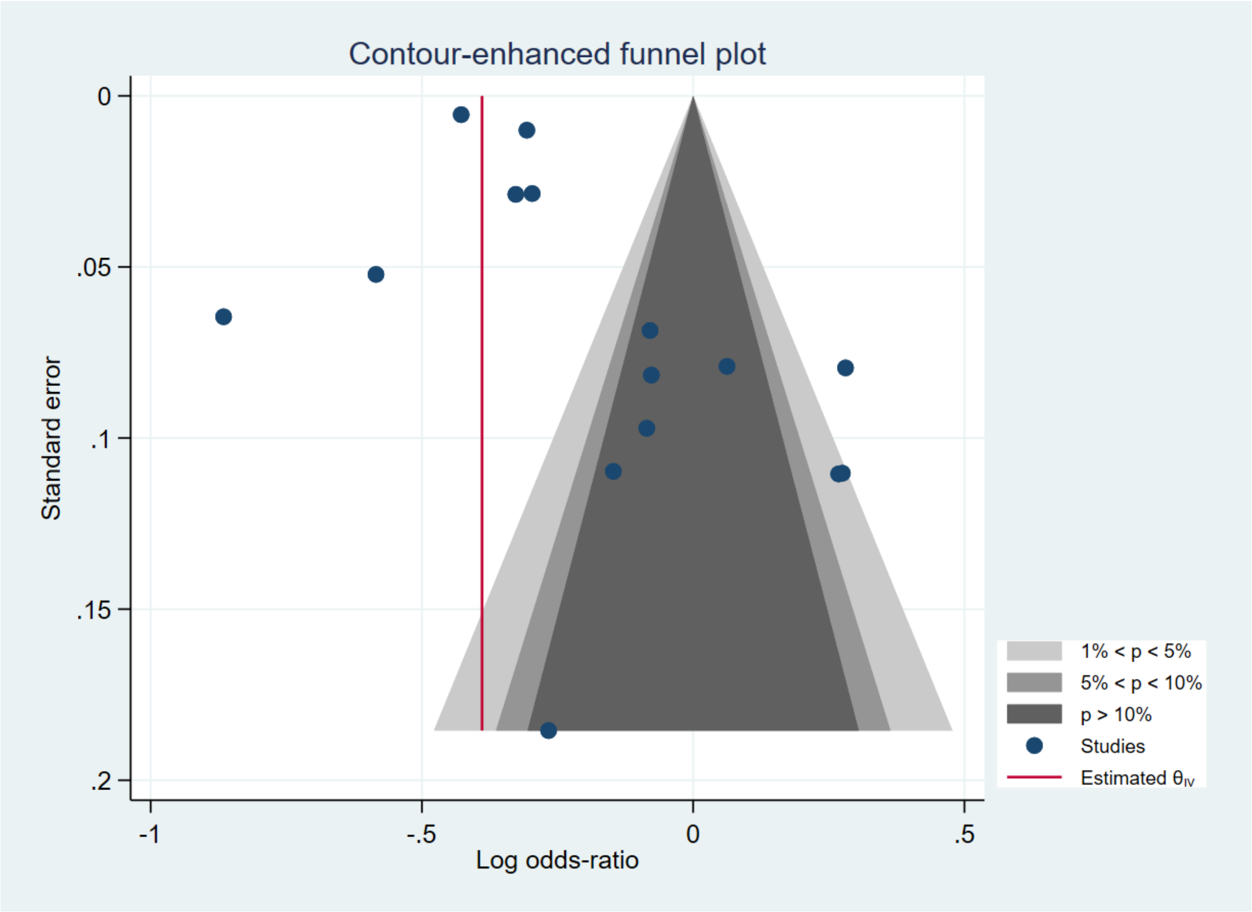

Supplementary Figure S4 Comparison between heavy and low alcohol consumption regarding incident AF. Sensitivity analysis.

Study	Heavy consumption AF	Heavy consumption No AF	Low consumption AF	Low consumption No AF	Log Odds-Ratio with 95% CI	Weight (%)
Djoussé L et al. 2004	237	951	163	692	0.06 [-0.17, 0.28]	24.16
Conen D et al. 2008	40	1,319	294	15,076	0.44 [0.11, 0.78]	18.11
Ariansen I et al. 2020	97	2,884	2,360	89,883	0.25 [0.04, 0.45]	25.06
Kim YG et al. 2020	14,753	657,968	116,123	4,900,595	-0.06 [-0.07, -0.04]	32.67
**Overall**					0.14 [-0.08, 0.35]	

Heterogeneity: $\tau^2 = 0.04$, $I^2 = 82.83\%$, $H^2 = 5.82$

Test of $\theta_i = \theta_j$: $Q(3) = 17.47$, $p = 0.00$

Test of $\theta = 0$: $z = 1.26$, $p = 0.21$

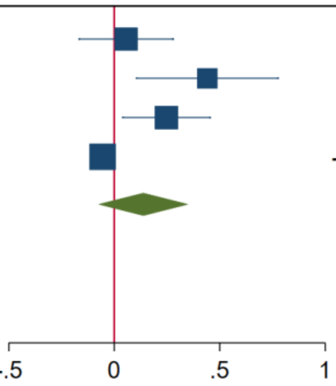

Random-effects DerSimonian-Laird model

AF: Atrial Fibrillation

Supplementary Figure S5 Comparison between moderate and heavy alcohol consumption regarding incident AF. Sensitivity analysis.

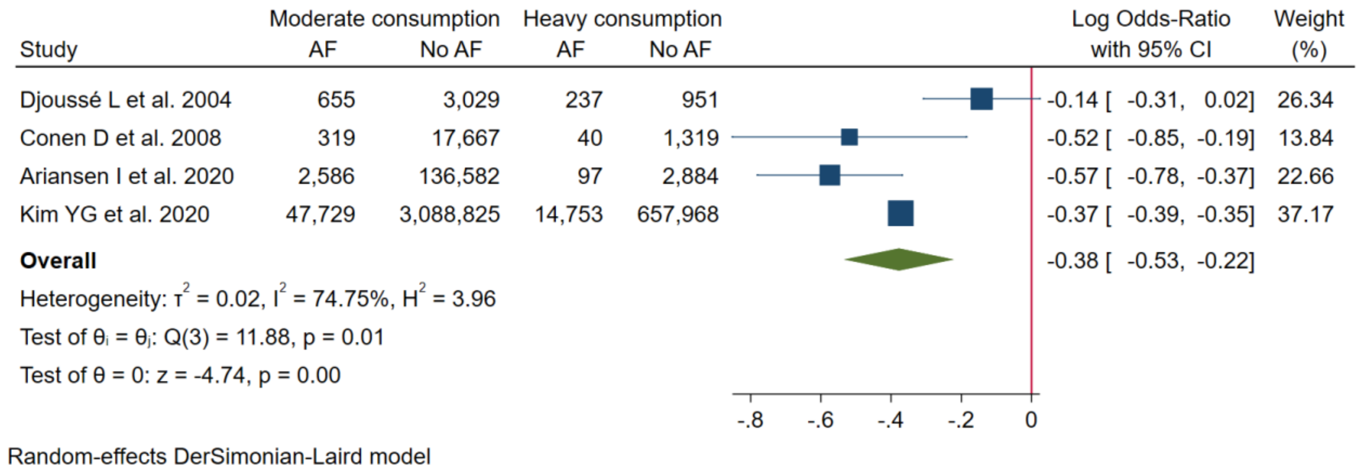

AF: Atrial Fibrillation

Supplementary Figure S6 Comparison between moderate and low alcohol consumption regarding incident AF. Sensitivity analysis.

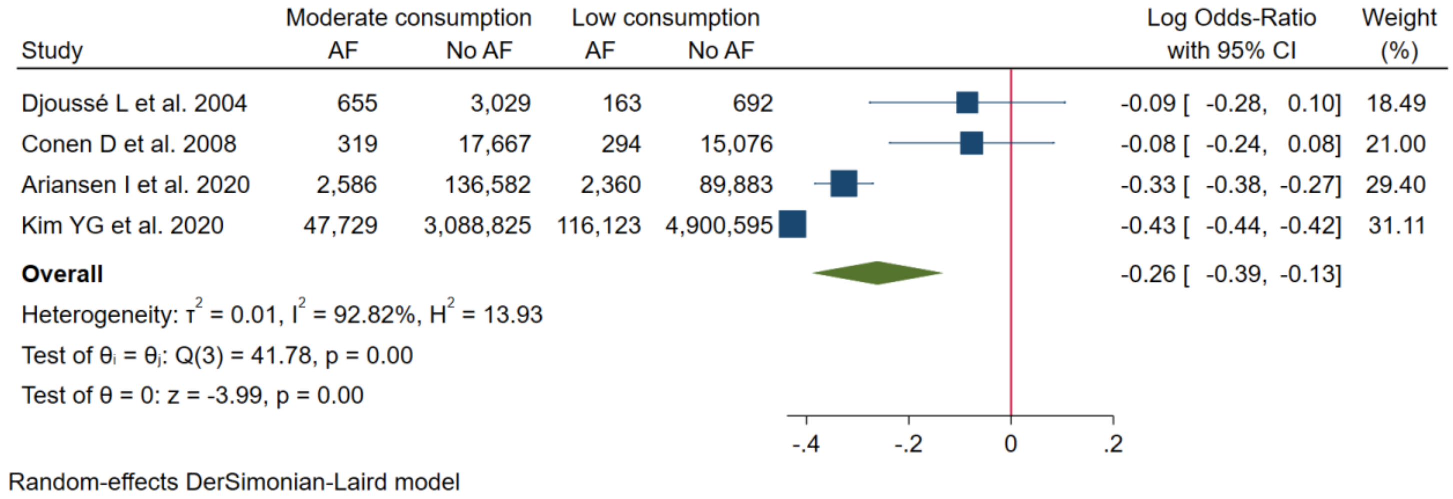

AF: Atrial Fibrillation
